# Decoding vesicle-based precision oncology in gliomas

**DOI:** 10.1093/noajnl/vdac035

**Published:** 2022-11-11

**Authors:** Syeda Maheen Batool, Tiffaney Hsia, Sirena K Khanna, Austin S Gamblin, Yulia Rosenfeld, Dong Gil You, Bob S Carter, Leonora Balaj

**Affiliations:** Department of Neurosurgery, Massachusetts General Hospital, Boston, Massachusetts, USA; Department of Neurosurgery, Massachusetts General Hospital, Boston, Massachusetts, USA; Department of Neurosurgery, Massachusetts General Hospital, Boston, Massachusetts, USA; Department of Neurosurgery, Massachusetts General Hospital, Boston, Massachusetts, USA; Department of Neurosurgery, Massachusetts General Hospital, Boston, Massachusetts, USA; Department of Neurosurgery, Massachusetts General Hospital, Boston, Massachusetts, USA; Department of Neurosurgery, Massachusetts General Hospital, Boston, Massachusetts, USA; Department of Neurosurgery, Harvard Medical School, Boston, Massachusetts, USA; Department of Neurosurgery, Massachusetts General Hospital, Boston, Massachusetts, USA; Department of Neurosurgery, Harvard Medical School, Boston, Massachusetts, USA

**Keywords:** extracellular vesicles, glioma, liquid biopsy, plasma

## Abstract

Extracellular vesicles (EVs) represent a valuable tool in liquid biopsy with tremendous clinical potential in diagnosis, prognosis, and therapeutic monitoring of gliomas. Compared to tissue biopsy, EV-based liquid biopsy is a low-cost, minimally invasive method that can provide information on tumor dynamics before, during, and after treatment. Tumor-derived EVs circulating in biofluids carry a complex cargo of molecular biomarkers, including DNA, RNA, and proteins, which can be indicative of tumor growth and progression. Here, we briefly review current commercial and noncommercial methods for the isolation, quantification, and biochemical characterization of plasma EVs from patients with glioma, touching on whole EV analysis, mutation detection techniques, and genomic and proteomic profiling. We review notable advantages and disadvantages of plasma EV isolation and analytical methods, and we conclude with a discussion on clinical translational opportunities and key challenges associated with the future implementation of EV-based liquid biopsy for glioma treatment.

Extracellular vesicles (EVs) are small, membrane-bound nanoparticles released into the extracellular environment via cell shedding and non-apoptotic blebbing.^1^ EVs can be broadly divided into 3 subtypes by size: exosomes (30-100 nm), microvesicles (50-1000 nm), and oncosomes (1-10 µm)^2–4^ and further classified by their cargo.^5^ The biogenesis of EVs results in the encapsulation of DNA, RNA, proteins, and cytosolic compounds within the lipid bilayer, all of which maintain the native configuration from the cell of origin and thus can describe both the physicochemical and biochemical properties of the cell.^5^ The biological functions of EVs depend on the source cells. However, these membrane-bound nanoparticles play an essential role in cell-to-cell communication via delivering proteins, nucleic acids, and metabolites. In addition, EVs have been shown to regulate a number of cellular processes, including proliferation, apoptosis, and autophagy. Given their membrane-bound nature, EVs remain relatively stable in circulation, increasing the average lifespan of the encapsulated material. There is substantial evidence of glioma pathology represented in EVs circulating in biofluids like cerebrospinal fluid (CSF) and blood, highlighting their potential in liquid biopsy.^6,7^ Other analytes that can be isolated from biofluids include circulating tumor cells (CTCs), cell-free DNA, and circulating nucleic acids. However, the concentration of EVs isolated from patient biofluid is significantly higher and more likely to be stable in circulation. For instance, CTC isolation requires a starting input of a large volume of fresh blood followed by immediate processing due to rapid deterioration of cell viability. Plasma is a preferred sample type for EV biomarker discovery, given that blood collection is minimally invasive, compared to CSF collection, and unlike serum, plasma does not contain coagulation factors, which potentially confound glioma-specific EV isolation and analysis.^8^ Here, we review prevalent EV isolation, quantification, and characterization strategies with a focus on plasma-derived EVs from patients with glioma (Figure 1).

**Figure 1.**
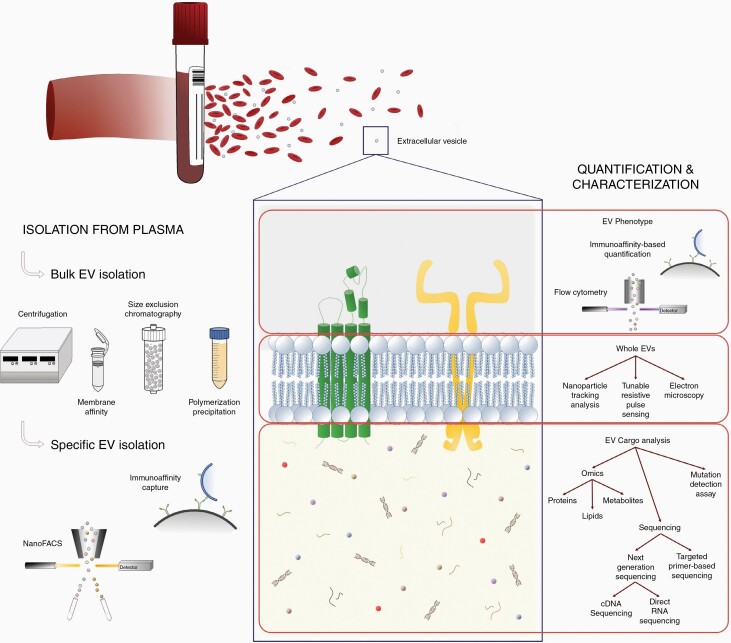
Summary of the main methods for isolation and analysis of glioma-derived extracellular vesicles circulating in patient plasma.

## Isolation and Analysis of Whole EVs

The current standards of EV isolation include bulk and specific isolation. Bulk isolation consists of centrifugation, membrane affinity, size exclusion, and polymerization precipitation strategies. Although successful at eliminating debris during isolation, they limit glioma-specific EV enrichment due to the heterogeneous healthy cell-derived EVs in plasma. It is crucial to differentiate tumor-specific EVs from this background. Specific EV isolation methods, such as immunoaffinity (IA) capture and nano-fluorescence activated cell sorting (nanoFACS) harness the EV phenotype to enrich glioma EVs. IA capture isolates EVs based on the expression of target surface proteins, such as EGFRvIII, which bind to antibodies conjugated to magnetic beads or surfaces.^9^ This in turn allows for isolation of glioma-derived EV subpopulations from plasma and downstream analysis for tumor-specific EV profiling.^10^ NanoFACS relies on target protein or cargo expression for EV isolation.^11^ Unlike IA capture, EVs remain in a single EV suspension for downstream analysis.^12^

Quantification and characterization of whole EVs are broadly categorized into total EV and EV subpopulation studies (Table 1). Analysis of total intact EVs rests on the delineation of total EV population size and concentration via techniques, such as nanoparticle tracking analysis (NTA) and tunable resistive pulse sensing (TRPS). These methods quantify particles based on the Brownian motion of nanoparticles in solution. As such, they are useful for detecting total EV populations. However, proteins and debris of similar sizes may also be detected, producing a nonspecific signal. More specific exploration of EV topographical information (size, shape, morphology) can be performed using transmission electron microscopy (TEM) and scanning electron microscopy (SEM). Analysis of EV subpopulations and single EV events, however, can be achieved using fluorescent labeling of EVs and measured using imaging flow cytometry (IFC) and fluorescence flow cytometry (FFC), as well as IA capture. Unlike total EV analysis, IFC and FFC analyze single events to determine phenotypic prevalence within single EVs and total concentration within the entire EV population.

**Table 1.** Analysis of Intact Extracellular Vesicles (EVs) From Plasma of Patients With Glioma

Biomarker	Method	Study
EV count, Annexin V	Cryo-electron microscopy (CM) Flow cytometry (FCM)	Evans et al^13^
EV count, Annexin V	FCM Electron microscopy (EM)	Koch et al^14^
EV count and characterization	Nanoparticle tracking analysis (NTA)	Cumba Garcia et al^15^
EV count and characterization	NTA Confocal laser scanning microscopy (CLSM)	Osti et al^16^
EV count and size	NTA Transmission electron microscopy (TEM)	Akers et al^17^
EV size and count, CD63, CD81, CD9	NTA TEM Imaging flow cytometry (IFCM) Correlative light electron microscopy (CLEM)	Ricklefs et al^18^
EGFR, EGFRvIII, PDGFR, PDPN, EphA2 and IDH1, R132H	Size and immunoaffinity Microfluidic nuclear magnetic resonance (µNMR) assay	Shao et al^19^
Total protein quantification	FCM TEM	Muller et al^20^

Enrichment, in both isolation and analysis, of glioma-specific EV subpopulations relies primarily on EV labeling. Labeling using fluorescent markers indicative of intact biological environments (ie, CFDA-SE) has been reported to quantify total EV populations in FC applications. Further analytical depth can be achieved via fluorescent conjugated antibodies for surface protein marker labeling, such as CD63 and CD9, for phenotypic characterization of glioma-derived EVs.^18^ Similarly, exploration of the cancer-specific EV landscape can be performed via labeling of glioma-related mutations, such as IDH1, EGFRvIII,^21^ and survivin.^22^ Although promising, this method of identification is burdened by antibody labeling standardization and processing. Recent studies have explored the use of endogenous fluorophores for the labeling of glioma-produced EVs. Utilizing the downstream features of 5-aminolevulinic acid (5-ALA, a photosensitizer used in fluorescence-guided surgery) metabolism, a subpopulation of EVs produced by 5-ALA-dosed malignant glioma tumors have demonstrated endogenous fluorescence via IFC.^23^ These fluorescent EVs have successfully been sorted from plasma background.^24^ The quantification of fluorescence intensity, EV concentration, and EV cargo from plasma provides a venue for EV-based glioma biomarker development.

Phenotype-based EV isolation and quantification methods are considered low throughput, but these modalities provide the highest specificity for glioma EV isolation and enrichment from a heterogeneous background for enhanced downstream analysis.

## Isolation and Analysis of EV Cargo

Once glioma-specific EVs are isolated and purified, downstream detection and functional characterization of cargo are achieved by a careful selection of the relevant method. The EV cargo is composed of multiple proteins (tumor-specific antigens, heat shock, transport, and immunogenic proteins) and cytosolic analytes, including nucleic acids (mRNA, lncRNA, microRNAs, and DNA), lipids, and metabolites.

### EV Nucleic Acid Detection

Extraction of the encapsulated nucleic acids (RNA, DNA) can be performed using a number of commercially available kits leading to a variation in the yield and size distribution. Of the available techniques, ExoRNeasy (Qiagen) represents the most efficient method of EV RNA extraction from a range of volume of patient plasma with minimal binding to ex-RNA-containing particles like ribonucleoprotein complexes.^25^ It is the most widely used platform for the extraction of purified EV RNA for downstream mutation detection and genomic interrogation. Inclusion of a standard extraction protocol improves reproducibility and design of large-scale validation studies (intra- and inter-institutional).

Polymerase chain reaction (PCR) methods represent one of the earliest methods of targeted mutation detection using patient-derived plasma. Both real-time and digital PCR methods rely on dye-based fluorescent quantification of cDNA and/or EV DNA via reagents such as the, most commonly using SYBR Green or TaqMan, of cDNA and/or EV DNA. Droplet digital PCR (ddPCR), a more recent platform employs an ultrasensitive fluorescent technique, which has enabled absolute quantification of mutant events in partitioned samples (>10,000 droplets) based on Poisson’s distribution. This approach has several advantages compared to qPCR: measurement of low abundance transcripts, tolerance to PCR inhibitors, less dependence on reference genes, higher signal-to-noise ratio, and higher sensitivity, thereby making digital bioassays a more reliable tool for detection of rare glioma-specific mutations.

IDH1 mutation is a key molecular alteration in gliomas and a noninvasive diagnosis via plasma-based assays will have many clinical applications. The mutant and wild-type IDH1 sequences in extracted EV DNA have been previously detected using the PCR platform.^26^ Similarly, ddPCR has been used successfully to detect TERT promoter mutations in EV DNA with a sensitivity of >70%.^27^ EGFRvIII is another important mutation that serves as a reliable diagnostic marker to distinguish glioma from healthy states.^28^ Studies have reported assays to detect this mutation in EV-derived mRNA from plasma (Table 2). However, the reported sensitivity and specificity have limited its translation in clinical settings. Overall, detecting these mutations in plasma has allowed disease monitoring, surveillance, and tailored treatment approaches. Different miRNA signatures characteristic of glioma have been proposed to allow disease stratification and monitoring of tumor burden over clinical course.

**Table 2.** PCR and Sequencing Studies on DNA and RNA Biomarkers From Plasma-Derived Extracellular Vesicles (EVs) in Patients With Glioma

Biomarker	EV Cargo Analyte	Method	Study
IDH1G395A	DNA	Conventional PCR Fast COLD-PCR	García-Romero et al^26^
TERT promoter	DNA	Droplet digital PCR (ddPCR)	Muralidharan et al^27^
PD-L1	DNA	ddPCR	Ricklefs et al^29^
EGFRwt, EGFRvIII	mRNA	Semi-nested PCR	Manda et al^30^
EGFRvIII	mRNA	Herringbone microfluidic device (EVHB-chip) ddPCR	Reátegui et al^9^
24 immune response and glioma progression-related genes (TIMP-1, IL-8, TGF-β, PD-1, etc.)	mRNA	Real-time quantitative reverse transcription PCR (qRT-PCR)	Muller et al^20^
miR-21, miR-103, miR-24, and miR-125	microRNA	qRT-PCR	Akers et al^17^
miR-210, miR-185, miR-5194, and miR-449	microRNA	qRT-PCR	Tabibkhooei et al^31^
54 GBM-specific differentially expressed genes	RNA	Nextera XT kit (Illumina) HiSeq 2000	Reátegui et al^9^
CD9, CD63, and CD81	RNA	Quantitative PCR (qPCR)	Ricklefs et al^18^

Genetic profiling of exosomes has elucidated details on the genomic and epigenetic landscape of gliomas. The presence of certain RNA populations, namely mRNAs, miRNAs, and lncRNAs, has been demonstrated using PCR methods. Recently, however, high-throughput transcriptome analysis of EVs has led to the discovery of diverse RNA species, including snRNA, snoRNA, piRNA, scRNA, and SRP-RNA, and their role in mediating the biological effects of EVs on recipient cells.^32^ However, most of these studies report results based on next-generation sequencing (NGS) of serum and not plasma.^33^ Currently, plasma is the preferred medium for the isolation and analysis of tumor-specific analytes despite the risk of clotting at room temperature, which increases the risk of EV lysis and degradation.^34^

Unlike conventional PCR, NGS allows detection and monitoring of both known and unknown molecular alterations.^35^ The majority of library preparation protocols, however, do contain a PCR amplification step with specific primers to improve the sensitivity of detection and quantification of low-level EV analytes. Additionally, library preparation kits have been tailored for size selection of short vs long RNA fragments.^25^ Medium length (60-300 nt) RNA sequencing requires the use of kit-free protocols. Common sources of bias in sequencing include adaptor dimers in ligation technique, size selection after cDNA synthesis, choice of sequencing platform, and subsequent bioinformatics analysis.^25^ Validation of obtained results using additional methods (PCR, Western blot, etc.) can reduce bias and improve robustness.

Given the availability of novel high-throughput methods, it is important to explore the potential use of these technologies for plasma-derived EV cargo transcriptomic and genomic interrogation. Compared to cDNA sequencing, direct RNA sequencing (Oxford Nanopore Technologies [ONT]) offers many opportunities: low input requirement (<1 µg), elimination of PCR bias, detection of ultra-long RNA fragments, and identification of isoforms, gene fusions, and novel transcripts.^36^ It can also elucidate the role of RNA modifications in gliomagenesis and their interactions with key molecular alterations (IDH, PTEN, MGMT, TERT). For instance, m^6^A methylation can now be detected and mapped using MeRIP-seq, a technique that combines NGS with m^6^A-methylated RNA immunoprecipitation.^37^

EV cargo represents a complex composition of RNA populations of varying lengths. However, a comprehensive overview of different classes of RNA encapsulated in EVs and their functional significance is still lacking. Due to the enrichment of small RNAs in EVs, most studies have focused on miRNA and small non-coding RNA. While most of the RNA is fragmented, little is known about the longer fragments present and their role in glioma progression. This population has not been previously explored due to the inherent limitations of conventional library preparation kits, namely low-depth coverage and low precision of sequencing. ONT, therefore, represents a promising platform for a more comprehensive analysis of coding, non-coding, and regulatory RNA populations in plasma-derived EVs.

### EV “Omics” Profiling

Protein components of EVs have been cataloged using a number of mass spectrometry-based modalities (Table 3). It is crucial to consider the influence of isolation protocol and physicochemical properties on proteome content. Western blotting is an immunodetection technique based on affinity binding of a primary and fluorescently labeled secondary antibody to a specific surface antigen in lysed EVs.^38^ Using this, a study measured the expression of tropomyosin kinase receptor (TrkB) in plasma-derived exosomes and its correlation to aggressiveness and gliomagenesis.^39^ Furthermore, key cytokines (IL-8, IL-10, IFN-γ) have been similarly identified and shown to be dysregulated in gliomas.^15^ Some limitations of this method include inability to multiplex, requirement of a large input of EV protein, and limited reproducibility.^40^

**Table 3.** Proteomics-Based Analysis of Biomarkers From Plasma-Derived Extracellular Vesicles in Patients With Glioma

Biomarker	Method	Study
Syndecan-1 (SDC1), 12 proteins differed in HGG vs LGG	Liquid chromatography-mass spectrometry (LC-MS)) Ultrasensitive proximity extension immunoassay (PEA) Enzymelinked immunosorbent assay (ELISA)	Chandran et al^41^
Tropomyosin kinase receptor (TrkB)	Western blot	Pinet et al^39^
GFAP, TAU	Dielectrophoresis(DEP) Immunofluorescence staining (IF)	Lewis et al^42^
INFγ, IL-10, IL-13, CD80, CD86, B7-1, B7-2, flotillin-1, ICOSL	Cytokine and checkpoint molecules arrays Western blot ELISA	Cumba Garcia et al^15^
11 differentially expressed proteins	Sequential window acquisition of all theoretical fragment ion spectra mass spectrometry (SWATH-MS) LC-MS	Hallal et al^43^
von Willebrand factor (VWF)	LC-MS	Sabbagh et al^44^
VWF, APCS, C4B, AMBP, APOD, AZGP1, C4BPB, Serpin3, FTL, C3, and APOE	Liquid chromatography-electrospray ionization-mass spectrometry (LC-ESI-MS)	Osti et al^16^
Fatty acid synthase (FASN)	Western blot Imaging flow cytometry (IFCM)	Ricklefs et al^45^

Another approach utilizes integrated immuno-based microfluidic isolation and protein analysis. Microfluidics devices allow fluorescent antibody-based detection of EVs on a chip rather than on a membrane or magnetic beads,^46^ allowing for isolation of a broader spectrum of EV antigens and potential biomarkers.^47^

Mass spectrometry (MS) analysis can be used for global and/or targeted proteomics. The general principle involves digestion of extracted proteins followed by the separation of peptides using gel-based (1D/2D gel electrophoresis) or gel-free platform (liquid chromatography). We can therefore deduce quantity and sequence details. Global (discovery-driven) proteomic approach achieves ionic selection either based on prevalence (data-dependent acquisition [DDA]) or predefined mass range (data-independent acquisition [DIA]).^48^ Targeted (hypothesis-driven) proteomic analysis is mostly conducted using multiple reaction monitoring (MRM), which allows parallel monitoring of up to a hundred predetermined peptides at different retention times.^48^ This approach has several advantages: improved sensitivity and specificity, ability to multiplex, and low input requirement.^49^ The existing studies have highlighted a few candidate proteins, however, a more extensive correlation study between exosome protein levels and glioma cell of origin is needed to delineate disease specific from exosome-enriched proteins.^50^ To fully harness the clinical potential, we need candidate markers to differentiate between low-grade and high-grade glioma. Syndecan-1 (SDC1), an exosome protein, represents an important example in this application, with the mRNA expression levels measured by MS and enzyme-linked immunosorbent assay (ELISA) were shown to be significantly different in GBM (glioblastoma) vs low-grade glioma cohort.^41^

No studies have investigated plasma EV-derived lipids or metabolites as putative biomarkers in glioma.

## Future Directions

EV-based liquid biopsy has tremendous clinical potential in establishing a minimally invasive and cost-effective platform for characterizing tumors using circulating analytes. However, despite the significant progress in this field, it is yet not recognized as a standard of clinical care. There are a number of factors to consider including the presence of technical and biological variability in preanalytical and analytical stages as outlined in Table 4. The development of multi-analyte tests will further improve the feasibility of using this platform to decipher the tumor genotypic and phenotypic landscape. Lastly, the collaboration between the public and private sectors is essential to improve standardization and reproducibility. With considerable clinical implications, it can be successfully employed as a rapid, reliable, noninvasive clinical decision making tool.

**Table 4.** Challenges of Extracellular Vesicle (EV) Isolation and Analysis and Proposed Recommendations

Method	Challenges	Recommendations
Isolation of EV cargo	Poor consistency among studies and highly variable isolation protocols Lack of predetermined handling and storage conditions Purification of EV preparations Consideration of the confounding patient-related and environmental variables	Use of optimized and standardized protocols Standard storage protocols Inclusion of strategies to remove potential contaminants (use of RNase, DNase, proteinase treatment) Careful selection of patient population and controls to minimize the influence of external variables (eg, age-dependent clonal heterogeneity)
Mutation detection (PCR)	Low input analyte Limited reproducibility Choice of blood component Low sensitivity and specificity	Use of ultrasensitive modalities with a lower mutant allele frequency (MAF) detection Large-scale validation studies (intra- and inter-institutional collaboration) More consistent and frequent use of plasma (vs serum) Methods to remove heterogeneous background and reduce the signal-to-noise ratio
Sequencing	Size selection bias (eg underrepresentation of medium size RNA) GC content bias Adapter dimers in ligation-based library preparation kits Lack of reproducible and standard bioinformatics pipelines Variability secondary to use of different sequencing platforms Limited reproducibility and clinical translation of findings (novel biomarkers)	Serial extractions of different-sized populations from the same patient sample. Careful selection of purification kit based on the population of interest. Comparison of different extraction protocols Modification of the kits to reduce ligation bias Use of approved and standard databases for mapping to reduce variability, reliable statistical tools, consistent normalization methods, inclusion of reference genes Use of identical sequencing technologies for accurate inter-study comparisons Validation using reliable techniques (PCR, Western blot, etc.)
Proteomics	Variability in isolated EV populations Limited proteome sequence coverage Paucity of information on glioma-specific protein mutations and protein-protein interactions Delineation of glioma-specific EV proteins from non-tumor markers	Minimize confounding variables (patient-related) and tailor the isolation protocol Use of high-throughput and accurate MS-based methods for a limited quantity of isolated proteins Proteogenomics; integrated approach incorporating targeted proteomics and RNA-seq data Development of sensitive and robust targeted proteomics in combination with downstream validation studies for functional characterization
